# Chemoprevention effect of the Mediterranean diet on colorectal cancer: Current studies and future prospects

**DOI:** 10.3389/fnut.2022.924192

**Published:** 2022-08-04

**Authors:** Asma Ismail Mahmod, Shatha Khaled Haif, Ayah Kamal, Israa A. Al-ataby, Wamidh H. Talib

**Affiliations:** ^1^Department of Clinical Pharmacy and Therapeutic, Applied Science Private University, Amman, Jordan; ^2^Department of Pharmacy, Princess Sarvath Community College, Amman, Jordan

**Keywords:** Mediterranean diet, inflammation, colorectal cancer, chemoprevention, natural compounds

## Abstract

Colorectal cancer (CRC) is the third most common cancer and the second most deadly cancer worldwide. Nevertheless, more than 70% of CRC cases are resulted from sporadic tumorigenesis and are not inherited. Since adenoma-carcinoma development is a slow process and may take up to 20 years, diet-based chemoprevention could be an effective approach in sporadic CRC. The Mediterranean diet is an example of a healthy diet pattern that consists of a combination of nutraceuticals that prevent several chronic diseases and cancer. Many epidemiological studies have shown the correlation between adherence to the Mediterranean diet and low incidence of CRC. The goal of this review is to shed the light on the anti-inflammatory and anti-colorectal cancer potentials of the natural bioactive compounds derived from the main foods in the Mediterranean diet.

## Introduction

**FIGURE 1:**
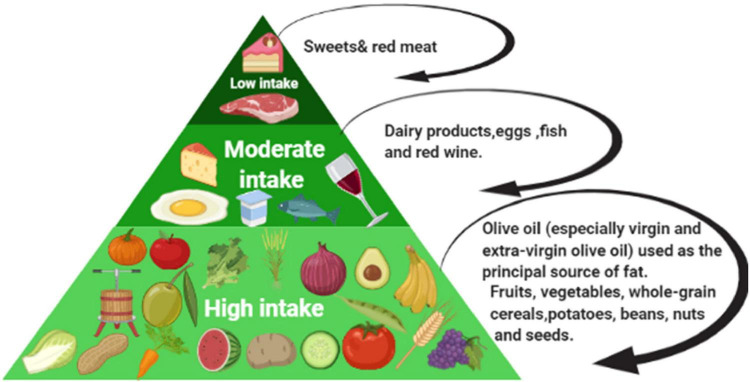
The Mediterranean diet intake level.

The Mediterranean diet (MD) are one of the many studied and well-known dietary pattern worldwide, and it has been associated with a broad range of benefits for health as well. Besides, the MD appears as the best diet pattern to reflect many characteristics of an ideal healthy diet. The roots of the traditional MD pattern are seen in civilizations encircling the Mediterranean Sea; historically, some of the 22 countries bordering the Mediterranean Sea. So that this pattern has been closely associated with the social behaviors and lifestyles of that region (1). The traditional MD is arranged from a high intake of plant foods (fruits, vegetables, pieces of bread and other cereals, potatoes, beans, nuts, and seeds); or fresh fruits as a typical dessert, and olive oil as the vital source of fat, reaching to a low intake of foods like red meat and sweets containing sugars or honey. The health benefits of individual foods and components of the MD (e.g., extra-virgin olive oil and nuts) have been well-documented (Figure 1) (2, 3). The benefits of the MD are not due to exclusively one component, but it is the whole food pattern as well as the wide range of traditional cuisine and lifestyle (4). Many factors are associated with the positive outcomes of the MD, such as traditional cooking methods, fasting practices, unique recipes, and using home garden vegetables (5). As well, the MD has been subject to many changes that shaped the current form today, those changes including culture, religion, agriculture production, climatic conditions, poverty, and economy (5, 6). According to the United Nations Educational, Scientific and Cultural Organization (UNESCO), the MD is recognized as “a set of traditional practices, knowledge, and skills passed on from generation to generation and providing a sense of belonging and continuity to the concerned communities” (7). The most consistent and robust evidence for the health benefits of MD has been observed in cardiovascular conditions, type 2 diabetes, metabolic syndrome, obesity, cancer, cognitive decline, and many others (8, 9). In observational studies, higher adherence to the MD was inversely associated with different types of cancer, including breast, colorectal, head, neck, respiratory tract, bladder, and liver (10).

Colorectal cancer (CRC) is the third most familiar malignancy and the second most deadly cancer. It has been reported about 1.9 million incidence cases and 0.9 million deaths worldwide in the year 2020 (11). There is evidence of higher CRC risk in Westernized society whose behaviors are characterized by a more elevated consumption of red and processed meat than in people living along the Mediterranean coast, who have a decreased overall cancer mortality correlated to their eating habits such as MD (12). Chronic intestinal inflammation, such as Crohn’s disease and ulcerative colitis, is predisposed to CRC (13). In addition, the upregulation of proinflammatory factors such as cyclooxygenase-2 is observed in inflammatory bowel disease-related CRC. The capacity for the MD to prevent CRC is likely due in part to the total anti-inflammatory effects exerted by the diverse food components that contribute to this dietary pattern, specifically those foods and beverages contributing a significant load of phenolic compounds (i.e., olive and fish oil, and plant-based foods) (14).

This review aims to summarize the most recent clinical and preclinical studies of the main micronutrients included in the components of the MD and to verify the correlation between their anti-inflammatory, gut microbiome modulation, and chemopreventive effects in CRC.

## Factors associated with the development of colorectal cancer

### Impact of genetic abnormalities on colorectal cancer initiation and progression

**FIGURE 2:**
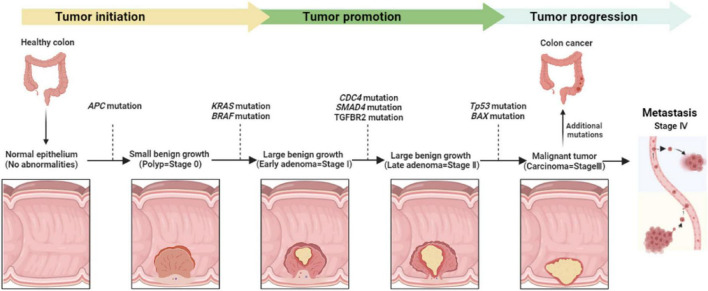
Colorectal cancer development, stages, and the main genetic modifications all along tumor progression.

Colorectal cancer can be promoted when intestinal epithelial cells are exposed to different genetic and epigenetic modifications that make them hyperproliferative (15). There are several distinct molecular pathways that modulate the progression of adenoma-carcinoma sequences, including chromosomal instability, microsatellite instability, and CpG island methylation (15–18). Hence, chromosomal abnormalities may associate with mutations that occur in particular oncogenes or tumor suppressor genes like *APC, KRAS, PIK3CA, BRAF, SMAD4*, or *TP53* (19). Besides, when mutations happened in DNA mismatch repair genes this is known as microsatellite instability which is found in 10–15% of sporadic CRCs (20). Moreover, DNA CpG methylation is involved in the early stage of CRC development (21) and associated with *BRAF* and *KRAS* mutations as well as *MLH1* methylation (22). CRC development begins from normal cells changed to hyperplastic polyp and then converted to sessile serrated adenomas ending up with cancer (Figure 2) (23). As a result, CRC is classified into five stages: stage 0 (benign polyp), stage I (tumor invades the muscularis propria), stage II (tumor invades tissue in the serosa), stage III (involved of visceral peritoneum), and stage IV (metastasis) (Figure 2) (24).

### Colorectal cancer and inflammation

In humans, up to 20% of all cancers result from chronic inflammation and persistent infections (25). CRC can be categorized as either sporadic, with inflammation following cancer onset, or colitis-associated CRCs induced by chronic inflammation. Both inflammatory bowel diseases, ulcerative colitis, and Crohn’s disease have a clear correlation with a significantly increased CRC risk, indicating chronic inflammation’s role in carcinogenesis (26). CRC that comes from inflammatory bowel disease (IBD) is responsible for about 2% of CRC mortality yearly. Despite optimal medical treatment, the chronic inflammatory condition associated with IBD raises the chance for high-grade dysplasia and CRC, along with the effect of the genetic and environmental risk factors and the microbiome (27).

**FIGURE 3:**
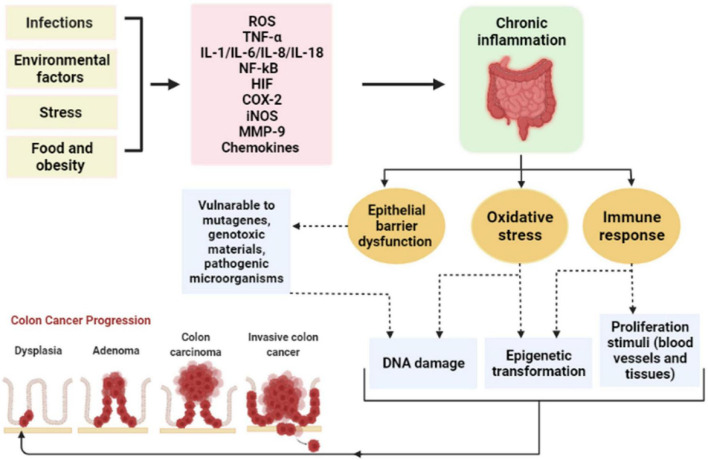
The correlation between chronic inflammation and the development of CRC. ROS, reactive oxygen species; TNF-α, tumor necrosis factor-alpha; IL, interleukin; NF-kB, nuclear factor kappa B; HIF, hypoxia-inducible factor; COX-2, cyclo-oxygenase-2; iNOS, inducible nitric oxide synthase; MMP-9, matrix metalloproteinases-9.

Several studies have demonstrated the strong correlation between chronic inflammation and tumorigenesis. Chronic inflammation could be prompted by: infections (viruses and bacteria), environmental factors (smoking and pollution), dietary factors, stress, and obesity (28). Besides, inflammation plays a role in tumor development, which mediates epigenetic alteration and modulation of oncogenes expression, DNA damage induced by oxidative stress and mutagens, as well as unrestricted tissue regeneration and proliferation (16, 29). In particular, chronic intestinal inflammation triggered different signaling pathways that augment tumor initiation and progression in CRC (Figure 3) (16). On the other hand, significant data have been associated between unbalanced gut microbiota and gastrointestinal tumorigenesis (30). The function of the intestinal epithelial barrier is affected by both chronic inflammation and microbial pathogens (31). They expanded gut permeability leading to ease in the translocation of microbial substances and stimulating an immune response (30, 31).

### Colorectal cancer and microbiome

The gut microbiota is categorized into commensal and pathogenic bacteria. There are four distinct groups of bacteria found in the gut microbiota, including *Firmicutes, Bacteroidetes, Actinobacteria*, and *Proteobacteria* (32, 33). Healthy gut microbiota plays an essential role in keeping the immune system active and able to attack opportunistic bacteria through particular receptors (e.g., Toll-like receptors) or gut microbiota’s metabolites (e.g., short-chain fatty acid) (34). Dysbiosis is the state of an imbalanced microbiome which can lead to a wide range of digestive problems such as irritable bowel syndrome (IBD) and CRC. An unhealthy gut microbiome can initiate inflammation and modify several signaling pathways resulting in the carcinogenesis of CRC (35). However, there are specific bacterial strains associated with the development of CRC, including *Fusobacterium nucleatum*, *Escherichia coli*, and *Bacteroides fragilis* (32). Tumorigenesis effects of dysbiosis can be summarized as the following: the genotoxicity (DNA damaging and mutations) effect of some bacteria and their metabolites, disrupting the gut surface permeability, which may promote inflammation, and modulation of the immune response (16, 32). Several studies have shown that modulation of the gut microbiome can improve the prognosis, treatment, and prevention of CRC (32).

## Selected components of the Mediterranean diet and their effect on reducing colorectal cancer risk

### Extra virgin olive oil: Phenolic compounds

Olive oil (OO) is an essential component of the MD with high nutritional values due to the presence of various bioactive compounds (36). Simple phenols, fatty acids, flavonoids, lignans, hydrocarbons, triterpenes, and phytosterols are the main chemical compositions of olive oil (37). Moreover, extraction methods play a critical role in determining the natural nutrients in (OO). Thus, applying cold extraction methods will produce extra virgin olive oil (EVOO), which is recognized with high phenolic content and low free fatty acids (38, 39). Extra virgin olive oil is known for its protective effect on CRC, conquers intestinal inflammation, and improves gut microbiota (40–42).

Hydroxytyrosol (3,4-DHPEA) and its secoiridoid derivatives are the main polyphenolic compounds in EVOO, and it originated from the hydrolysis of oleuropein during the ripening of olives (43). Hydroxytyrosol (HT) is known for its diverse pharmacological effects, including antitumor, anti-inflammatory, immunomodulatory, antimicrobial, and neuroprotective potential (44–48). Recently, Hydroxytyrosol has been tested in mice subjected to dextran sulfate sodium (DSS)-induced colitis (49). The study revealed a high potency of (HT) in suppressing inflammation and alleviating colitis symptoms via downregulation of IL-6, IL-1β, TNF-α, and myeloperoxidase enzyme. Moreover, hydroxytyrosol was able to reduce NLRP3 inflammasome expression, and thus suppressed the expression of IL-18, IL-1β, and caspase-1 in DSS-induced ulcerative colitis (50). It also improved oxidative biomarkers and downregulated colon malondialdehyde, myeloperoxidase, and NO levels along with a significant reduction in mortality rate and disease activity index of albino rats with induced ulcerative colitis (51).

Besides the anti-inflammatory effects of (HT), it is also known to have anticancer properties and is involved in cancer hallmarks modification and tumor regression effect (52, 53). Hormozi et al. reported that (HT) induced apoptosis via upregulation of the caspase-3 gene and increased BAX/Bcl2 ratio in a human CRC cell line (LS180 cells) (54). It enhanced the expression of the antioxidant enzymes, which emphasized the antiproliferation effect (54, 55). As well, another anticancer mechanism of hydroxytyrosol was the inhibition of thioredoxin reductase 1(TrxR1) enzyme and promoted G1/S cell cycle arrest (48). It is noteworthy that a high level of (TrxR1) enzyme has been detected in CRC cells, which correlated with poor prognosis and chemotherapy response (56, 57). Moreover, hydroxytyrosol repressed the growth of human colorectal adenocarcinoma cells (HT-29) in both models *in vitro* and *in vivo* through downregulation of epidermal growth factor receptors (58). As well, it increased the expression of the CRC -associated-1 gene (COLACA1) in the same cell line leading to reduce tumorigenesis and an upraised survival rate (59).

Oleuropein is the ester form of hydroxytyrosol with β-glucosylated elenolic acid. It is found in olive leaves and EVOO with different content, and it gives olives a bitter and pungent taste (39, 60). Oleuropein has been involved in many pharmacological applications due to its properties, such as antioxidant, anti-inflammatory, and antineoplastic properties (61, 62). Oleuropein exhibited an anti-inflammatory effect via the suppression of inflammatory mediators, including NF-Kβ, COX-2, caspase-3, TNF-α, and Inos (61, 63, 64). Besides, it conquers inflammation by inhibiting of MAPK/NF-κB signaling pathway (65) and reduces the expression of IL-6, TNFR60, TNFR80, and ICAM-1 (66). In a recent study, Motawea et al. suggested that oleuropein was effective in reducing the following pro-inflammatory cytokines: IL-1β, TNF-α, IL-10, COX-2, iNOS, TGF-β1, MCP-1, and NF-κB in an induced colitis rat model (67). Besides, nanostructured lipid carrier-oleuropein was tested in the DSS-induced colitis experimental model and it exhibited a modulation of the inflammatory biomarkers via decreasing the level of TNF-α, IL-6, and hindering neutrophil infiltration (68). As well, oral intake of a diet supplemented with olive cream and probiotics revealed a synergistic anti-inflammatory effect in DSS-induced chronic colitis (69).

Previous studies also demonstrated the anticancer effect of oleuropein, including cell proliferation and migration inhibition, apoptosis induction, and growth signals modulation (39, 62, 70). Cárdeno et al. suggested that oleuropein significantly improved apoptotic mediators and decreased HIF-α expression in human CRC (HT-29 cells) (71). Besides, oleuropein repressed the activity of the main transcription proteins, including NF-κB, STAT3, PI3K/Akt, and β-catenin in AOM/DSS-induced CRC mice (72).

### Tomato: Lycopene

Vegetables are consumed in the MD abundantly, and tomatoes are one of the universal MD components in the countries of the Mediterranean basin (73). Tomatoes are the edible fruits of the tomato plant (*Solanum lycopersicum*) that belongs to the *Solanaceae* family (74). The consumption of tomatoes has been related to a low incidence of chronic degenerative diseases, and various types of cancer (74, 75). These health benefits are expected to be associated with the presence of a wide range of phytochemicals, including carotenoids (lycopene and β-carotene), and polyphenolic compounds (flavonoids, flavanones, and flavones). Besides, high concentrations of other nutrients such as vitamin A, ascorbic acid, potassium, and folate have been reported in the chemical composition of tomatoes (73, 76). According to the chemical structure of the carotenoids, they are classified into carotenes (purely hydrocarbons such as lycopene and β-carotene) and xanthophylls (having oxygen in their structure such as lutein, zeaxanthin, and β-cryptoxanthin) (77, 78). Lycopene (LC) is a lipid-soluble pigment of natural carotenoids, which could be found in fresh tomatoes and processed tomato products (79, 80). Moreover, it is responsible for the red color of many fruits and vegetables like tomatoes, pink grapefruit, red guava, and watermelons (77). Many epidemiological studies have shown the biological activities of lycopene, including antioxidant, anti-inflammatory, cardioprotective, and anticancer effects (73, 81, 82).

From an anti-inflammatory perspective, lycopene was able to reduce the following inflammatory biomarkers: TNF-α, IL-1β, and IL-6 in the acetic acid-induced ulcerative colitis rat model (83). As well, it has significantly suppressed the level of NF-kB, TGF-β1, and caspase-3 along with upregulation of GSH expression and catalase activity in the same experimental model (84). A recent study has shown the effect of lycopene on colitis progression by lowering the disease activity index score, improving the colon length, and rising the expression of catalase, GSH-Px, and SOD (85).

On the other hand, several studies have described the antiproliferation activity of lycopene in CRC cells. Lin et al. have shown the chemo-preventive effect of lycopene in human colon cancer cells (HT-29). It downregulated the MMP-7 expression and hindered tumor development and tumor cell invasion (86). In a mouse xenograft model, LC decreased the expression of PCNA and β-catenin proteins, which are associated with tumor growth and progression. As well, lycopene attenuated the level of MMP-9 in tumor-bearing mice (87). MMPs are known to enhance the tumor microenvironment and promote cell invasion resulting in poor prognosis and low survival rate in CRC patients (88).

### Herbs and spices

Medicinal plants have remained the primary source of medications; many of the pharmaceuticals that are now accessible were derived from them, either directly or indirectly. Many plants have been shown to have vital roles in the treatment and prevention of various illnesses in various regions of the globe (89, 90). The bioactive phytochemical elements of many plants have traditionally been employed in Asian medicine (91, 92). Health care services are provided by the traditional healer plants, which are founded on religious and cultural backgrounds, knowledge, attitudes, and beliefs (90, 91, 93). Recent years have seen a surge in interest in assessing plant foods and discovering phytochemicals with the potential to inhibit carcinogenesis.

#### Onion: Quercetin

Onions (*Allium cepa*) are members of the Liliaceae (94). The Liliaceae family has around 250 genera and 3700 species (95–97). *Allium cepa* is one of the world’s oldest and most frequently grown vegetables, growing in practically every climate zone, from tropical to cold temperate (98). Although *A. cepa* is referred to as the “Queen of the Kitchen,” it is distinguishable by the color of its outer scales (yellow, red, or white), its taste (sweet or bitter), and whether it is consumed fresh or powdered (99, 100).

Onion is roughly 90% water, with a significant concentration of nutritional fiber and carbohydrates. In terms of vitamins and minerals, onion has a low salt level while being rich in vitamin B6, vitamin C, folic acid, and minerals (Ca, Fe, S, Se, Mg, Ph, and k) (101–103). On the other hand, has a low lipid content and a pool of free amino acids (104, 105). Onions are high in a range of phytochemicals with beneficial properties, including organosulfur compounds, phenolic compounds, polysaccharides, and saponins (106–113). Sulfur compounds such as DATS, diallyl disulfide (DADS), ajoene, and sallylmercaptocysteine (SAMC), onionin A (114). Two flavonoid subgroups are abundant in onion: anthocyanins, which give certain kinds of their Reddish-purple color, and the primary pigments are flavonols, which include quercetin, which is found conjugated as quercetin 4′–*O*-glycopyranoside, quercetin 3,4′–O-diglycopyranoside, and quercetin 3,7,4′–O-triglycopyranoside (115–117). Along with quercetin, additional flavonols found in onions include kaempferol, luteolin, and isorhamnetin (118, 119).

Onion bulbs are used not only for their taste and aroma but also for the nutritional value they provide to the human diet. Several studies have demonstrated that onion and its bioactive components have a variety of pharmacological effects (120), including anti-inflammatory (121), anti-obesity (121), anti-spasmodic agent (100, 122), anticancer (123–125), and wound healer (126). Additionally, it has long been recognized as a helpful therapy for a variety of medical conditions including diabetes (95, 127), cardiovascular disease (128), hypertension (106), anxiety (129), and asthma (130). Furthermore, *A. cepa* demonstrated that it suppressed gram-positive bacteria more efficiently than gram-negative bacteria (131) and that it reduces DNA damage and breaking owing to the presence of quercetin, a potent antioxidant (109, 132).

The onion bulb extract has been shown to both prevent and reverse colitis by modulating several pro-inflammatory signaling pathways, including the mechanistic target of rapamycin (mTOR), the mitogen-activated protein kinase family (MAPK), cyclooxygenase-2 (COX-2), and tissue inhibitors of metalloproteinases (TIMP), as well as several molecules involved in the apoptotic pathway (121, 133, 134). They also found several phytochemicals such as saponins, tannins, and anthocyanin can help fight inflammation (104, 105). In the MC3T3-E1 preosteoblastic cell line, quercetin in a dose-dependent manner strongly inhibited the nuclear factor kappa-light-chain-enhancer of activated B cells (NF-jB) (135). Furthermore, in animal and human investigations, quercetin lowered TNFa/IL-10 and IL-8/IL-10 ratios (136–138). Umoh et al. showed that red onion may reduce inflammation by inhibiting NF-jB, MARK, and STAT-1, perhaps via the action of its active component quercetin (139). Besides, quercetin was able to reduce inflammation in DDS-induced colitis mice via upregulation of GSH levels (140).

In recent years, scientists have concentrated their efforts *in vitro*, *in vivo*, and human investigations on the prevention of cancer through diets with a high percentage of onion. Inhibiting cell cycle, triggering apoptosis, inhibiting tyrosine kinase, regulating p53 protein, and inhibiting antioxidant activity that interferes with several phases of cancer cell creation, development, differentiation, and metastasis (117, 139, 141–145). Onion extracts or their key bioactive constituents have shown strong anticancer activity against prostate, stomach, breast, lung, colorectal, laryngeal, and esophageal cancers, pancreatic, adenocarcinoma, and glioblastoma (146, 147). Quercetin, a novel onion component, might cause G (2) phase arrest, reduce colon cancer cell growth, and trigger autophagic cell death (148). In particular, it has been detected the endocannabinoid receptor (CB1-R) expression, PI3K/Akt/mTOR pathways, and the pro-apoptotic JNK/JUN pathways in Caco2 and DLD-1 cells. It was found a considerable increase in the expression of the endocannabinoid receptor (CB1-R), as well as suppression of important survival signals including PI3K/Akt/mTOR (145). The administration of quercetin was obsessively monitored for 48 h. In both the Colo-320 and Colo-741 cell lines, there was an increase in BAX immunoreactivity after quercetin treatment, but only in the Colo-320 main cell line was there existing a substantial reduction in Bcl-2 immunoreactivity (149). By a remarkable mechanism, quercetin (5 M) was able to significantly reduce the migratory and invasive potential of Caco-2 cells, resulting in decreased MMP-2 and MMP-9 expressions, whereas *E*-cadherin was downregulated. furthermore, quercetin has been shown to inhibit the production of inflammatory mediators such as TNF-α, COX-2, and IL-6 (150). Male Wistar rats were given (200 mg/kg, 28 days) or (0.5 g, 27 weeks) of onion-rich of quercetin had a big impact on ACF formation, mucin depletion, mitosis, and increasing the apoptosis percent in the treatment group. Although significant influence on cell proliferation and the expressions of p53 and BAX (151). (152) demonstrated the quercetin-loaded MPEG–PCL nanomicelles (Qu-M) dispersed entirely in water and released quercetin for a long time *in vitro* and *in vivo*. *In vitro*, Qu-M enhanced apoptosis induction and inhibited cell proliferation in CT26 cells. Furthermore, the mice (BALB/c) subcutaneous CT26 colon cancer model was constructed to assess the therapeutic effectiveness of Qu-M in greater detail. Qu-M investigates a high impact on cell death, preventing tumor angiogenesis, and limiting cell proliferation (152). Supplying quercetin (30 mg/kg, 4 weeks) to AOM/DSS-induced colon cancer mice (Wild-type C57BL/6J mice) decreased the number and size of tumors by a significant margin including, reduce the inflammation produced by AOM/DSS, recovered leukocyte numbers, also reduces oxidative stress indicators such as lipid peroxide (LPO), nitric oxide (NO), superoxide dismutase (SOD), glucose-6-phosphate (G6PD), and glutathione (GSH) (153).

#### Garlic: Allicin

Garlic or *Allium sativum* L. is a bulbous plant of the Alliaceae family that grows in the Mediterranean region (154, 155). In addition to its medicinal properties, garlic is also widely used as a food and spice (156, 157). Garlic is distinguishable from other members of the allium family by its clove-shaped bulbs and flat leaves (158, 159). Garlic includes at least 33 sulfur compounds, various enzymes such as peroxidase, allinase, and myrosinase, 17 amino acids, and minerals such as selenium, calcium, copper, iron, potassium, magnesium, and zinc, as well as vitamins A, B1, and C, fiber, and water (156, 160–163). It has the highest concentration of sulfur compounds of all Allium species (158, 163). Sulfur compounds are responsible for garlic’s strong odor as well as many of its therapeutic properties (163). Saponins (proto-eruboside B, eruboside B, sativoside), lectins, and flavonoids are some of the other elements found in this plant (159, 160). A compound called allicin is one of the most important biologically active compounds. Garlic does not have allicin until it is crushed or cut. The enzyme allinase, which breaks down alliin into allicin, is activated when the garlic bulb is damaged. Once it’s made, it quickly breaks down, but the speed of this reaction changes depending on the temperature. Allicin can still be found in garlic that has been refrigerated for a few days. At room temperature, it breaks down into smelly sulfur compounds like diallyl di- and tri-sulfides, ajoene, and vinyldithiins in just a few hours (164–168).

Garlic was highly prized in ancient Egyptian, Greek, and Chinese cultures as a food and medicinal (169, 170). It has been studied clinically for a variety of illnesses, including hypertension, hypercholesterolemia, diabetes, common cold, and cancer prevention (171–178). This plant has antibacterial, antifungal, antioxidant, immune system stimulant, and anti-parasitic properties (159, 162, 176). Garlic’s medicinal potential has also been investigated in a variety of inflammatory illnesses, including allergic rhinitis, allergic asthma, IBD, rheumatoid arthritis, and atherosclerosis (158, 177). Breath odor is a common adverse effect of using garlic, both orally and intravenously (162, 179).

A person’s risk of developing malignant tumors may be increased or decreased depending on the amount of some foods consumed or the number of others omitted from their diet. On this premise, dietary treatments are regarded to have the capacity to prevent or modify malignancies. One of these natural compounds, garlic (*Allium sativum*), has been studied for medicinal purposes. Allicin, a compound found in garlic, has been shown to inhibit CRC metastasis by strengthening the immune system and limiting the growth of tumor arteries (180).

Using allicin, an active ingredient derived from the popular seasoning agent or condiment *Allium sativum* L., was able to reduce the secretion of pro-inflammatory factors such as interleukin-6 (IL-6), prostaglandins (PG), nitric oxide (NO), interleukin-1 (IL-1), interleukin-6 (IL-6), and TNF-a, while simultaneously increasing anti-inflammatory cytokines such as IL-10 (181–184). Rats with Acrylamide (AA)-induced intestinal damage were used to examine the possible therapeutic benefits of allicin food supplementation. Allicin significantly reduced the expression of Toll-like receptor 4 (TLR4), NF-kB signaling pathway proteins, and proinflammatory cytokines in AA-treated rats by boosting the synthesis of SCFAs (185). In Caco-2 cells, allicin (25 mg/ml) can significantly inhibit p-38 and the JNK pathway activation. Allicin also suppressed the production of TNF-α and IL-6 generated by IL-1 at the mRNA and protein level in a dosage-dependent manner (186). According to Li et al. (186), an oral dose of allicin enhanced the colonic histopathology score and investigated the synergistic effects of allicin (30 mg/kg)-mesalazine (30 mg/kg) and allicin (30 mg/kg)-sulfasalazine (100 mg/kg) on TNBS (50 mg/kg) induced Wistar rats. Therapy with allicin-mesalazine decreased the colonic histopathology score from 5.83 to 2.10, whereas treatment with allicin sulfasalazine decreased it to 3.38. TNF-a levels were lowered by allicin-mesalazine therapy to 2.65 (pg/ml) from 6 (pg/ml), while allicin or mesalazine treatment alone reduced them to roughly 3.8 (pg/ml). TNBS therapy decreased IL-4 concentration to less than 4 (pg/ml), while mesalazine-allicin treatment increased their concentration to 5.76 (pg/ml), but neither allicin nor mesalazine alone could boost their expression to synergistic levels.

It was investigated the impact of allicin on cell proliferation in colon cancer cell lines HCT-116, LS174T, HT-29, and Caco-2 *in vitro*, as well as the underlying processes. Allicin has been demonstrated to have chemo-preventive effects on critical cellular processes such as mitochondrial membrane potential maintenance, intracellular redox control, and cell division. Allicin triggers G2/M arrest alters intracellular glutathione (GSH) levels (187) and causes a transitory decrease in intracellular GSH levels (187). Allicin treatment caused apoptotic cell death in HCT-116, as shown by increased hypodiploid DNA content, reduced levels of B-cell non-Hodgkin lymphoma-2 (Bcl-2), increased levels of BAX, and increased capacity to release cytochrome c from mitochondria to the cytosol. Allicin also caused NF-E2-related factor-2 (Nrf2) to be translocated to the nucleus of HCT-116 cells. Although Allicin’s cytotoxic effects were considerable when evaluated in four distinct human colon cancer cell lines (188). Experiments on animal models of carcinogenesis showed that components of garlic (e.g., allyl sulfides) suppress both the start and promotion phases of tumorigenesis in a wide range of malignancies including colorectal, lung, and skin (189). Perez-Ortiz et al. (190) Test the efficacy of a thiosulfinate-enriched garlic extract in combination with 5-fluorouracil (5-FU) or oxaliplatin chemotherapy in colon cancer cells as a new chemotherapy regimen that may also lower the cost of clinical treatment. The cytotoxic effects of an *Allium sativum* extract enriched in thiosulfate were investigated in two distinct human colon cancer cell lines, Caco-2 and HT-29, respectively. The doses of allicin (43–60 g/mL) were discovered to substantially decrease colon cancer cell growth and induce apoptosis. The impact of Allicin on the azoxymethane/dextran sodium sulfate (AOM/DSS) CRC mice model on STAT3 is being studied. Through various ligand-mediated phosphorylation, STAT3 plays key roles in cytokine signaling pathways, as well as cell proliferation and death. STAT3 activation causes the transcription of target genes such as Bcl-2, Bcl-xL, Mcl-1, and p21, all of which are important in cell survival and proliferation (180, 191). STAT3 activation may increase cell proliferation, angiogenesis, and inhibit apoptosis in human cancer cells. According to the western blot results, Allicin reduced the levels of phosphorylated STAT3. Allicin also inhibited the expression of Mcl-1, Bcl-2, and Bcl-xL. Therefore, allicin may be able to prevent colonic carcinogenesis in AOM/DSS mice *in vivo* (180). As an example, Diallyl trisulfide (DATS), an organosulfur compound isolated from garlic, has shown anticancer activity both *in vitro* and *in vivo* by reducing tumor mitosis and enhancing histone acetylation of H3 and H4 in both tumors and healthy cells (168).

#### Oregano: Carvacrol

Oregano is one of the most highly prized spices in the world, both commercially and culinary (192). oregano is formed from the terms “Oros” and “Ganos,” which both refer to the beauty of the mountains in ancient Greek (193). Oregano is the common name for at least 61 different species spread over 17 different genera and six different families (194). The family Lamiaceae contains the genus *Origanum*, which is the primary source of well-known oregano spice (194). All of the other plant families (Rubiaceae, Scrophulariaceae, Apiaceae, and Asteraceae) play a minor role (194–196). Monoterpenes and sesquiterpenes make up the majority of the essential oils in the Lamiaceae family. Their action is linked to the presence of carvacrol and thymol, which are combined with the primary elements of oregano, *p*-cymene, and terpinenes (196–198). Also, Oregano has a high nutritional value since it includes considerable quantities of vitamins and minerals while having a low salt level (195, 199, 200). Numerous studies have demonstrated the beneficial effects of oregano on human health, including its use in the treatment of a wide range of ailments, including respiratory tract disorders, gastrointestinal disorders (anti-stomachic and tonic agent), as an oral antiseptic, analgesic, urinary tract disorders (as a diuretic and antiseptic agent), anti-inflammatory, and even anticarcinogenic properties (201–205).

Oregano plant species have been extensively utilized in traditional medicine to treat inflammation-related disorders via a variety of mechanisms, including Reduced synthesis of proinflammatory cytokines such as TNF-α, IL-1, and IL-6, and increased production of anti-inflammatory cytokines such as IL-10 (206), as well as inhibition of aldose reductase and lipoxygenase (206, 207). Oregano, which is high in essential oils, has been shown to inhibit the COX-2 enzyme, which is linked to tissue inflammation (208). Carvacrol may also play a role in reducing the side effects of chemotherapy, in addition to its anticancer properties. The anticancer medication irinotecan hydrochloride causes a condition in which inflammation and cell damage are triggered by the transient receptor potential cation channel subfamily A, member 1 receptor. Carvacrol is an agonist of this receptor leading to an effective reduction of inflammation biomarkers, such as nuclear factor b and cyclooxygenase 2, as well as oxidative stress, as measured by glutathione, malondialdehyde, and NOx levels in a mouse model of inflammatory arthritis (209).

There are a lot of different Flavonoids and phenolic compounds in species of oregano, and some of them have been shown to fight colon cancer (210). As a result, research has looked into whether oregano flavonoids and phenolic compounds could be used as anti-colon cancer.

The “shutting down” of many cancer survival pathways, including the ERK/MAPK and PI3K/Akt pathways, may be responsible for the overall suppression of colon cancer cell growth following treatment with *Origanum syriacum* ethanol extract (211). The whole extract of *Origanum vulgare* is responsible for the apoptosis-inducing action (212). Carvacrol significantly slowed the growth, migration, and invasion of colon cancer cells by stopping cells at the G2/M phase and causing them to die, reduced Bcl-2 expression, phosphorylated extracellular regulated protein kinase (p-ERK) and p-Akt, and increased BAX and c-Jun N-terminal kinase (p-JNK) expression (LoVo and SW620 metastatic cells line) (213). Showcase of Mexican oregano in colon cancer cells, there was an increase in the expression of BAX (apoptotic protein) and a decrease in the expression of Bcl-2, PARP, and Survivin (anti-apoptotic proteins), as well as an increase in the expression of caspase-3 in various oregano cultured plants (wild type, *in vitro* and *ex vitro* plant tissue culture) (202). Thymol, another phenolic component typically found in *O. syriacum*, was discovered to suppress the growth of bladder and colon cancer cells (HCT116, LoVo, and Caco2 cells line) *in vitro*, which was promising (214, 215). The key mechanistic activity of thymol’s action was identified to be the inhibition of JNK and p38 as the main mediators (214, 215). In another investigation, oregano aqueous infusion had the strongest radical scavenging efficacy in HT29 cells. Oregano’s strong antioxidant activity has been linked to a variety of substances including carvacrol and other phenolic compounds (202).

#### Saffron: Crocin

Saffron, the world’s most expensive botanical spice, commonly known as “red gold,” is made from the dried stigmas of *Crocus sativus* L., a member of the broad Iridaceae family (216, 217). It is an autumn-flowering, high-value, low-volume spice crop plant that originated in the Middle East and is now farmed in China, India, Iran, Azerbaijan, Turkey, Egypt, Morocco, Greece, Spain, Italy, France, and Mexico (218–221). In addition to the elements that already contained in saffron, such as protein, fiber, lipids, vital minerals, and vitamins B1 and B2 (222–224). There are various key metabolite components, including carotenoids (crocetin, crocin, zeaxanthin), monoterpene aldehydes (picrocrocin and safranal), monoterpenoids, and phenolic compounds (anthocyanins and flavonoids) that contribute to the diverse pharmacological effects of this substance (216, 225–227). Three major bioactive chemicals (crocin, picrocrocin, and safranal) are found in significant amounts among these metabolites, and they are responsible for the Safran’s distinctive red color (crocin), bitter taste (picrocrocin), odor, and aroma (safranal), and other characteristics (146, 228, 229).

Saffron consumption correlates with a lower risk of many types of cancer (227, 230–232), improvement of depression and memory loss (226, 227), regulation of menstruation (233), accelerated wound healing in burn injuries, and relief of cough and asthmatic breathing (229, 230). It has also been shown to be an antihypertensive (225, 234), antianxiety (233), insulin resistance lowering agent (233, 235), cardioprotective (236), and gastroprotective properties (233).

Various research has shown that the anti-inflammatory and antioxidant effects of saffron constituents are due to their significant inhibitory effects against cyclooxygenase 1 and 2 enzymes and prostaglandin E2 production (237), attenuating endoplasmic reticulum stress signaling, blocking pro-inflammatory cytokine production such as TNF-α, inhibiting transcription factors such as nuclear factor kappa B (NF-kB), which exacerbates chronic inflammation, and suppressing inflammatory gene expression (223, 238). Additionally, the anti-inflammatory properties of saffron derivatives affect neuroinflammation (237). Following the study (239), the researcher concluded that crocin protects rat gastric mucosa ethanol-induced injury through the expression of anti-inflammatory, antioxidant, antiapoptotic, and mucin secretagogue mechanisms, which are most likely mediated through increased PGE2 release. After only 4 weeks, Kawabata et al. found that crocin feeding could prevent Dextran Sulfate Sodium (DSS)-induced colitis and decrease TNF-α expression, IL-1β, IL-6, IFN-γ, NF-kB, COX-2, and iNOS in the colorectal mucosa and increased nuclear factor (erythroid-derived 2)-like 2 (Nrf2) expression (240). Concerning the effectiveness of saffron in the treatment of ulcerative colitis (US), studies have shown that oral administration of crocetin to mice (25–100 mg/kg per day) for 8 days significantly ameliorated TNBS-induced UC, as evidenced by a reduction in NO, neutrophil infiltration, and lipid peroxidation in the inflamed colon, favorable expression of TH1 and TH2 cytokines, and down-regulation of the NF-kB (241).

According to the literature, saffron and its components have chemopreventive activity via the inhibitory effect of saffron on cellular DNA and RNA synthesis, modulation of lipid peroxidation, antioxidants, immune modulation, enhancement of cell differentiation, inhibition of cell proliferation, modulation of carcinogen metabolism, cell cycle arrest through p53 dependent and independent mechanisms causing apoptosis, the interaction of carotenoids with topoisomerase II (227, 242–247). While the majority of *in vivo* and *in vitro* studies focused on isolated bioactive compounds from saffron, Aung et al. (248) revealed that *C. sativus* and its primary ingredient, crocin, effectively reduced the proliferation of CRC cell lines (HCT-116, HT-29, SW-480) and non-small cell lung cancer cell lines (NSCLC) by MTS test while not affect normal cells. Aung et al. (248) demonstrated that saffron crudes and its main compound crocin can be used to supplement current CRC treatments by limiting cancer cell proliferation and motility progression by targeting the Metastasis-Associated in Colon Cancer 1 (MACC1) as a major causal metastasis-inducing gene. Crocetin (0.8 mmol/L) significantly triggered cell cycle arrest and P21 induction and caused cytotoxicity in SW480 cells by increasing apoptosis and lowering DNA repair capability in a time-dependent manner (249). Another research revealed that long-term intraperitoneal injection of crocin (400 mg/kg body weight) improves survival and inhibits tumor development in female rats with colon cancer generated by subcutaneous injection of rat adenocarcinoma DHD/K12 PROb cells (227). Crocin exhibited antiproliferation activity against the HCT116 cell line via induction of apoptosis and attenuation of the ratio of p-STAT3/STAT3 (250). As well, it suppressed tumor growth in colitis-associated CRC mouse model via modulation of the Wnt/PI3K pathway. Crocin was also able to lower disease-activity index and mucosal ulcer inflammation by regulating antioxidant markers, including catalase (CAT) activity and malondialdehyde (MDA) (251).

#### Rosemary: Rosmarinic acid

*Rosmarinus officinalis* (Rosemary) is a typical houseplant growing around the globe that belongs to the Lamiaceae family (252, 253). The chemical composition of rosemary extract was examined to determine its active principles, which indicated the existence of many compounds, including rosmarinic acid (RA), caffeic acid (CA), chlorogenic acid, carnosic acid, rosmanol, and carnosol (252, 254–256). Therefore, three types of chemicals have been linked to the biological activity of *R. officinalis* L.: a volatile fraction, phenolic compounds, and di and triterpenes (254, 255).

Rosemary has a long history of usage in food to change and improve tastes. On the other hand, rosemary extracts have anti-inflammatory, antioxidant, antimicrobial, antitumor, antispasmodic, and anti-diabetic bioactivities. The low toxicity and strong cardioprotective, hepatoprotective, neuroprotective, diuretic effect, estrogenic effect, as well as memory enhancement and pain relief have been investigated in the reviewed literature (253, 257–262). On the other hand, rosmarinic acid (RA) was able to reduce inflammation in AOM/DSS-induced colon cancer mouse model by inhibiting NF-kB and STAT3 pathways (263). 264 suggested that RA alone or in combination with black rice extract can suppress colitis disease in DSS-induced colitis mice. The results of the study have shown a reduction in the inflammatory mediators expression, including IL-6, IL-1β, TNF-α, iNOS, and COX-2 (264).

**FIGURE 4:**
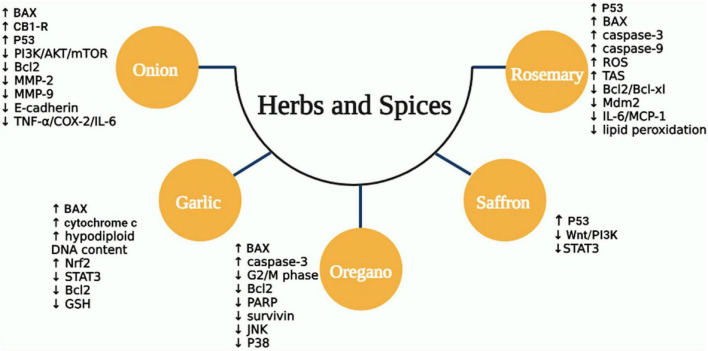
The mechanisms of anticancer activity for some types of MD herbs and spices. ROS, reactive oxygen species; TNF-α, tumor necrosis factor-alpha; IL, interleukin; COX-2, cyclo-oxygenase-2; MMP-9, matrix metalloproteinases-9; JNK, Jun N-terminal kinase; BAX, Bcl-2-associated X protein; PARP, poly adenosine diphosphate ribose polymerase; Nrf2, nuclear factor-erythroid factor 2; STAT3, signal transducer and activator of transcription 3; GSH, glutathione; CB1-R, cannabinoid receptor type 1; Mdm2, mouse double minute 2 homolog; MCP-1, monocyte chemoattractant protein-1; Wnt, wingless-related integration; PI3K, phosphoinositide 3-kinase.

Rosemary has been shown anticancer properties in both models: *in vitro* and *in vivo*. Several of these properties have been ascribed to its principal constituents, including carnosic acid, carnosol, ursolic acid, and rosmarinic acid (252, 265). Rosemary has been shown to have significant antiproliferative activity against several human cancer cell lines (266–269), induce apoptosis via nitric oxide production (270, 271), antioxidant activity (265, 266, 272), decreased TNF-α, IL-6, and COX2 levels (273, 274), suppress lipid peroxidation (261, 275, 276), prevent carcinogen-DNA formation (265), stimulation of p53 and BAX (277), reduction of Bcl-2, Mdm2, and Bcl-xL expression, and stimulation of caspase-3 and -9 expression (272, 274, 277, 278). Another probable method is via inhibiting Akt phosphorylation, which is required for cancer cell proliferation, growth, and survival (279). A study has tested the antiproliferation activity of rosemary extract on HT-29 and SW480 cells. Rosemary extract suppressed tumor cell growth via increasing intracellular ROS, reducing in G0/G1 phase, and improving cells accumulation in the G2/M phase (280). Moreover, Valdés et al. reported that carnosol-enriched extract showed significant inhibition of cell growth by inducing G2/M cell cycle arrest in colon cancer cells (SW480 and HT-29 cell lines) (281). A recent study suggested that rosmarinic acid (RA) can reduce the rate of adenocarcinoma formation in azoxymethane-induced CRC in rats. It upregulated the total antioxidant status (TAS) and decreased the expression of IL-6 and MCP-1 (282). Using male Wistar rats with DMH-induced colon cancer, RA exhibited a chemoprotective effect via suppressing tumor formation and decreasing lipid peroxidation (276). Figure 4 summarized the anticancer effect of herbs and spices in CRC.

### Whole grains and cereals: β-D-glucan

Whole grains are cereals that have the complete grain kernel (bran, germ, and endosperm) in contrast with refined grains that contain the endosperm only. There is considerable evidence that chronic diseases could be avoided by the consumption of whole-grain cereal products, which can reduce the risk of obesity, metabolic syndrome, type 2 diabetes, cardiovascular disease, cancer, and mortality from these chronic diseases (283, 284). Whole-grain cereals are an abundant source of fiber and bioactive compounds. For example, whole-grain wheat consists of 13% dietary fiber and at least 2% bioactive compounds excluding fiber. In the bran and germ fractions, still greater proportions are attained: about 45 and 18% of dietary fiber, and about 7% and at least 6% of bioactive compounds, respectively (285). The total dietary fiber of wheat ranges from 9 –to 20% and it consists of both soluble and insoluble portions. The two major components of dietary fiber in wheat are arabinoxylan (AX) and β-D-glucan (283). While barley and oats contain β-glucan as the primary fiber in the whole kernel, AX is present in much less content (286). As well, cellulose and hemicellulose are the major fiber components of corn bran and brown rice (287).

Dietary fibers provide numerous benefits, including a lower risk of cancer and enhanced colon health, Where low dietary fiber consumption has been linked to both local and systemic chronic inflammation (288). Recently, a study using a synbiotic composed of arabinoxylan (AX) and *Lactobacillus fermentum* HFY06 was tested to determine its ability to relieve DSS-induced colitis. AX and *L. fermentum* HFY06 inhibited the activation of the NF-κB signaling pathway, downregulated the mRNA expression levels of NF-kBp65 and inhibited the TNF-α, and exerted anti-colitis effects (289). As well, the short-chain fatty acids (SCFA), particularly butyrate, are byproducts of dietary fiber fermentation by certain microorganisms in the intestinal colon, and they exhibited anti-inflammatory actions on both gut epithelial and immune cells. Hence, inflammation signaling pathways involving nuclear factor kappa-B (NF-kB) and deacetylase are inhibited by SCFA (288, 290). Several studies have been established to investigate the anti-inflammatory effect of the major types of dietary fibers. Such as the beneficial effect of oat β-D-glucan has been tested on Sprague–Dawley rats with TNBS-induced colitis. The results showed a significant reduction in IL-6, IL-10, C reactive protein (CRP), and IL-12. As well, β-D-glucan reduces some selected inflammatory markers, including COX, PGE2, and thromboxane A2 (TXA2). The results indicate the therapeutic effect of dietary oat beta-glucan supplementation in colitis (291). In another study, oat beta-glucan has been tested also on male Sprague Dawley rats with TNBS-induced colitis. The results proved the in-direct antioxidant effect of beta-glucans by agonistic binding of immune cells to membrane receptors, which results in increased antioxidant response and removing systemic effects of colon inflammation (292).

Another well-known function of dietary fiber is to lower the CRC incidence, as it reduces the concentrations of carcinogens and procarcinogens in the feces. Furthermore, it shortens the residence time of carcinogens in the lower gastrointestinal tract, reducing their absorbance and contact time with colon epithelium cells (293). As previously stated, dietary fibers digested by intestinal bacteria produce SCFA which has a protective effect against the growth of cancer cells (294, 295). 296 examined the role of dietary fiber in polyposis by using TS4Cre × cAPCl°x 468 mice. The results showed that a high fiber diet significantly increased SCFA-producing bacterium as well as SCFA levels. This was associated with an increase in SCFA butyrate receptor and a significant decrease in polyposis. The prebiotic activity of fiber could be the key mechanism for the protective effects of fiber on colon carcinogenesis. Overall, the findings revealed that insoluble fermentable fiber may protect against CRC (296). As well, a dietary fiber (β-glucan) with quercetin anti-colonic cancer effect has been tested. The findings demonstrated that alternating β-glucan and quercetin consumption alleviated colon damage and reduced mortality in CRC mice, with a 12.5% reduction in mortality. Consumption of β-glucan and quercetin alternated dramatically reduced TNF-α, increased the relative frequency of Parabacteroides, and downregulated three genes linked to inflammation and cancer (Hmgcs2, Fabp2, and Gpt) (297). Moreover, β-glucan was tested in human colon cancer cells (SNU-C4) and it exhibited antiproliferation activity by reducing Bcl2 expression and upregulation of BAX and caspase-3 levels (298).

### Fish: PUFA/n-3 fats

Fish is another important component of the traditional MD diet. In recent years, there has been a lot of emphasis on the positive effects of fish eating, which has been reinforced by the idea that the ocean is a fantastic source of new compounds. Fish meat is abundant in anti-inflammatory n-3 polyunsaturated fatty acids (PUFA), such as eicosapentaenoic acid (EPA) and docosahexaenoic acid (DHA), which have been proven to reduce the risk of cardiac mortality by 30–45%, and lessen ischemic stroke incidence (299, 300). As well, n-3 PUFA appears to be protective against dementia and Alzheimer’s in the elderly (301). It has been proven to affect various types of cancer, including breast, ovarian, prostate, lung, skin, colon, colorectal, pancreatic, and stomach cancers. These benefits of n-3 PUFA are a result of their antioxidant, anti-inflammatory, anti-apoptotic, and neurotrophic properties (302). As recently reviewed, n-3 PUFAs have anti-inflammatory effects against inflammatory diseases, including IBD, psoriasis, and rheumatoid arthritis by lowering arachidonic acid (AA) proinflammatory activities, increasing the production of endocannabinoids with EPA or DHA in their structure and thus anti-inflammatory properties, lowering the production of inflammatory cytokines like IL-1, IL-6, and TNF-α, lessening in T-cell proliferation and the formation of IL-2 (303). Moreover, they increase the production of anti-inflammatory markers; e.g., soluble IL-6r, IL-10, transforming growth factor-β (TGF-β) and lowering the expression of adhesion molecules on immune cells and endothelium. As well, the homeostasis of tissues has been restored after inflammation as a result of n-3 PUFA metabolites production such as resolvins, protectins, and maresins, which act as specialized pro-resolving mediators. These pro-resolving bioactive lipids act as “stop-signaling” of the inflammatory response and have important anti-inflammatory and anti-carcinogenic properties, by increasing macrophage phagocytosis, efferocytosis, and leukocytes egress (304, 305).

Recently, the impact of n-3 PUFA and probiotics have been investigated in BALB/c mice subjected to 2,4-Dinitrobenzenesulfonic acid (DNBS) induced-chronic colitis. Administration of combination reduced the concentrations of inflammatory mediators such as IFN-γ, TNF-α, and IL-17A. The findings showed that the combined effect of probiotics and n-3 PUFA might have a protective effect against colon injury and inflammation by creating synergistic effects (306). Moreover, the anti-inflammatory effect of n-3 PUFA was investigated in a rat model of acetic acid-induced UC. The results showed that after administration of n-3PUFAs, the expression levels of IL-1 and Caspase-3 were downregulated, whereas Bcl-2 was upregulated. These findings imply that n-3 PUFAs protect the colonic mucosa of rats against acetic acid-induced UC, and may aid in the repair of UC via anti-inflammatory and anti-apoptotic properties, as well as a regenerative endogenous antioxidant mechanism (307).

Interestingly, n-3 PUFA is also known to have anticancer potentials and is associated with the alteration of cancer hallmarks and tumor regression activity. It can modulate cyclooxygenase (COX) metabolism and reduce the production of several prostanoids including prostaglandin (PG) E2 in tumors whilst possibly increasing the production of lipid mediators involved in the resolution of inflammation such as lipoxins and resolvins, which may have anti-cancer properties (308). As well, n-3 PUFA has been found to bind to the plasma membrane of cancer cells, changing the content and fluidity of the lipid membranes. This can cause signal transduction to be inhibited, reducing cancer cell viability and encouraging apoptosis (309). Other CRC-promoting signaling pathways, including the Wnt/ß-catenin pathway, the MAPK/ERK pathway, and the PI3K-PTEN system have been reported to be downregulated by n-3 PUFA (310). In a recent study on CRC cells, SW620 and HCT-116 parental and HCT-116 mutant cells isogenic for constitutively active PI3K were treated with free or ethyl esterified n-3 PUFA. The results showed the ability of n-3 PUFA ethyl esters to inhibit PI3K activity confers their potency to reduce CRC cell invasion, but not proliferation. (311) Furthermore, n-3 PUFAs can activate pro-apoptotic signaling by interacting with G protein-coupled receptors (GPCRs), resulting in an anti-CRC activity. Non-epithelial cells including adipocytes and macrophages have been found to express these GPCRs. Activation has the potential to change macrophage polarization and reduce inflammation, both of which are critical for n-3 PUFA anti-cancer action (312). Additionally, in the LS174T human CRC cell line, the effect of EPA derived from n-3 PUFAs on cell number, cell proliferation rate, and caspase-3 enzyme activity was studied. When EPA concentrations were increased, caspase-3 activity rose by 3.4 times relative to untreated control cells at 200 mol EPA and reduces the number of CRC cells and their growth rate (313). A further study examined the effect of DHA on migration in CRC cell lines and found that 100 mM DHA inhibited Granzyme B expression in three human CRC cell lines (HCT116, CSC4, and HT-8), limiting their capacity to undergo epithelial-mesenchymal transition (EMT) (314).

### Grapes: Resveratrol

Grapes have been associated with health benefits for many years, despite a lack of scientific evidence, and have been closely linked with diet since ancient times, particularly in Mediterranean countries. Several studies conducted around the world over the last two decades have shown that consuming grapes have beneficial impacts on antioxidant capacity, lipid profile, and the coagulation system (315).

Grape juice is made mostly from European (*Vitis vinifera*) and American grape species (*Vitis labrusca*). Both species have high levels of polyphenolic compounds, such as caffeic acid, gallic acid, *p*-coumaric acid, and stilbenes (*trans*-resveratrol). Resveratrol (RV) is one of the most important polyphenols found in grapes. As well as, Flavonoids such as quercetin, rutin, myricetin, catechin, and epicatechin (315, 316). The amount of RV in grapes varies by grape genotype, cultivar, growing season, and climatic factors. It can be found in the leaf, skin, bud, stem, seed, bud, and root. Regardless, the majority of it is found in the grape skin, with much less in the juice and wine. Grape skin and juice contain more glycosylated RV than free RV (317). The benefits of RV are numerous. The most well-known benefits include anti-inflammatory, anti-proliferative, and chemopreventive. Multiple lines of evidence from *in vivo* and *in vitro* laboratory research suggest that the anti-inflammatory properties of RV can be explained by preventing the synthesis of anti-inflammatory factors (318). Chronic inflammation is one of the main mechanisms involved in colon cancer. Therefore, the anti-inflammatory effect may be beneficial in the treatment of CRC.

Resveratrol has been shown *in vitro* to reduce the production of pro-inflammatory mediators such as IL-1 and IL-6, as well as to down-regulate both mRNA expression and protein secretion of IL-17 in a dose-dependent manner. Resveratrol is also associated with the inhibition of 5-lipoxygenase, cyclo-oxygenase-2 (COX-2), and Nuclear Factor-kB (NF-kB). As well, suppressing the expression of tumor necrosis factor α (TNF-α), interleukin 8 (IL-8), and interferon-γ (IFN-γ) will lead to a decrease in the ulcerative colitis process (319–321). Ren et al. investigated the anti-inflammatory effect of RV on HEK-293T cells and HeLa cells the results have shown that RV suppressed endogenous NF-kB activity and TNF-α induced NF-kB activation (322). Another study has demonstrated the anti-inflammatory activity of RV in the DSS-induced colitis mouse model. The results exhibited a reduction in IL-2, IFN-γ, GM-CSF, IL-1β, IL-6, KC/GRO, and TNF-α, along with a significant effect on gut microbiota by increasing the abundance of *Bifidobacterium* (323).

Besides the anti-inflammatory effect of resveratrol in grapes, it also has a high potential for inhibiting tumor initiation, development, and promotion. The majority of research on grapes’ cancer-preventive properties focuses on resveratrol (324). The anticancer activity of resveratrol is mediated by a variety of molecular mechanisms and signaling pathways. Such as activation of the mitochondrial and caspase cascade enzymatic systems, as well as death-induced cytokines and their receptors are upregulated, as are cyclin-dependent kinase inhibitors, and tumor suppressor genes. Additionally, resveratrol will lead to downregulation of survivin, cFLIP, cIAPs, and antiapoptotic proteins (Bcl-2 and Bcl-xL), all of which are linked to the development of chemoresistance. Although resveratrol suppresses tumor cell proliferation by activation of proapoptotic proteins (P21 and P53) and suppression of hippo–YAP, inhibition of MAPK, phosphoinositide 3-kinase (PI3K)/Akt, nuclear factor β (NF-β), activating protein-1 (AP-1) HIF-1α and signal transducer and activator of transcription 3 (STAT3) (325–327).

Several studies were conducted to investigate the effect of resveratrol on different cancer cell lines. Such as *in vitro* study on human CRC cells HCT116 and SW620, the results indicated that RV dose-dependently upregulated the expression of several proapoptotic proteins such as BAX, cytochrome *c*, cleaved caspase-9, and caspase-3, while anti-apoptotic protein Bcl-2 expression levels was reduced in RSV-treated CRC cells. Overall, resulted in the suppression of CRC cell viability, increased cell apoptosis, and ROS levels compared with the control group, as well as activated the mitochondrial apoptotic pathway (328). In another *in vitro* study of resveratrol on HCT116, a human CRC cell line, the treated cells showed cell growth inhibition and apoptosis induction, as well as downregulation of intracellular AKT1 and IL-6 expression (329). Moreover, an *in vitro* cellular model of aggressive and resistant colon cancer enriched in CSCs was chosen as doxorubicin-resistant LoVo/Dx cells (a subline of the LoVo cell line), which were treated with RV and celastrol. The results have shown that celastrol and resveratrol produce an antitumor activity against metastatic LoVo cells and cancer stem-like by inducing apoptosis and cell cycle arrest, by increasing *SIRT*1 gene expression, resulting in overcoming apoptosis resistance in LoVo colon cancer cells (330). Furthermore, a study using CRC-derived cell lines, LoVo and HCT116, found that resveratrol inhibited CRC invasion and metastasis by suppressing Wnt/-catenin signaling and, as a result, the expression of its target genes such as c-Myc, MMP-7, and MALAT1, which leads to the inhibition of CRC invasion and metastasis (331).

### Nuts: Hazelnuts/β-sitosterol

Hazelnuts (HN) produced by *Corylus avellana* L., a member of the genus Corylus of the Betulaceae family, are widely consumed around the world, and the common hazel is widely dispersed along the southern European coast and the Black Sea region (332). HN has several health benefits. Many studies showed the effect of HN on the reduction of LDL-C levels, and a tendency to lower total cholesterol accumulation. Besides, it reduced the incidence of certain chronic diseases such as cancers (333). The main chemical compositions of HN are simple phenols, lipids, and monounsaturated fatty acids, as well as, a source of minerals, tocopherols, tocotrienols, squalene, triterpenes, and phytosterols. The total phytosterol content of HN varies between 133.8 mg/100 g and 263 mg/100 g of oil. The most common is β-sitosterol (BS), which accounts for 83.6% of the total (334, 335).

β-sitosterol has immunomodulatory and anti-inflammatory activity, several studies suggested that BS can suppress inflammation through the NF-κB pathway (336, 337). Furthermore, BS stimulates the activity of T helper (Th) cells, as well as T cells and natural killer cells (338). A study investigated the effects of both stigmasterol and BS on DSS-induced colitis in C57BL/6J male mice. The results showed that both BS and stigmasterol significantly inhibited colon shortening, minimized fecal hemoglobin contents, and lowered the severity of colitis in the middle and distal colon. As well, they significantly suppressed the activation of the inflammatory master regulator NF-kB (339). Moreover, the effect of BS on 2,4,6-trinitrobenzene sulfonic acid (TNBS)-induced colitis in mice was also examined to see if it also exhibits anti-colitis properties. The study showed that BS inhibited colon shortening and resulted in lower macroscopic scores and myeloperoxidase activity. In the colons of TNBS-induced colitis mice, BS reduced the expression of proinflammatory cytokines TNF-α, IL-1, and IL-6, as well as COX-2 and activation of NF-kB. These data suggest that BS may help with colitis by suppressing the NF-kB pathway (340). Furthermore, in DDS-induced colitis in male C57BL/6 mice, BS was able to reduce the levels of TNF, IL-6, and IL-1 in intestinal tissue, indicating that β-sitosterol administration significantly reduced inflammatory damage to colonic tissues, including colon edema, crypt distortion, goblet cell loss, and mononuclear cell infiltration. These findings suggest that it could be useful in treating chronic colitis (341).

Besides the anti-inflammatory effects of BS found in hazelnuts, it has also been shown to protect against cancers such as breast, colon, colorectal, and prostate cancer. BS can halt tumor development and promote programmed cell death in cancer cells (342). A recent study tested the anticancer effect of BS-mediated silver nanoparticles (AgNP) on human colon cancer (HT-29) cells. The results suggested that BS improved apoptosis via inducing p53 expression in HT-29 cells (343). Furthermore, it inhibited the expression of breast cancer resistance protein (BCRP) and restored oxaliplatin (OXA) sensitivity in drug-resistant CRC cells. The study also found that BS could activate p53 by disrupting the p53–MDM2 interaction, resulting in increased p53 translocation to the nucleus and silencing the NF-κB pathway, which is required for BCRP expression. These findings showed that β-sitosterol can regulate CRC response to chemotherapy by mediating the p53/NF-B/BCRP signaling axis (344). Shen et al. (345) have reported that liposomal β-sitosterol can prevent tumor migration of colon carcinoma via downregulation of MMP-9 expression and modulation of Th1 immune markers. Additionally, BS, campesterol, and stigmasterol have been tested on colon cancer cells (Caco-2). The results revealed BS and other polyphenols induced reversible arrest in phase G_0_/G_1_ of the cell cycle (346). In HCT116 cells, BS induced apoptosis was accompanied by a decrease in anti-apoptotic Bcl-2 protein and mRNA and a concurrent rise in proapoptotic BAX protein and mRNA, as well as cytochrome c release from the mitochondria into the cytoplasm. The expression of cellular inhibitor of apoptosis protein-1 (cIAP1) was also suppressed by BS treatment (347). As well, the anti-CRC effects of BS were investigated in BALB/c nude mice. The study has proved that the treatment of mice with β-sitosterol decreased tumor growth by lowering PI3K/Akt expression, promoting Bad activation, decreasing Bcl-xl, and increasing cytochrome-c release, resulting in caspase-9 and caspase-3 activation, PARP cleavage, and apoptosis.

### Hot pepper: Capsaicin

Hot pepper (*Capsicum annuum* L.), usually called chili, is a diploid, facultative, self-pollinating crop that belongs to the Solanaceae family (closely related to the potato, tomato, eggplant, tobacco, and petunia). Hot pepper contains many essential vitamins, minerals, and nutrients that have a significant role in human health (348–351). Peppers are a rich origin of both vitamins C and E (352, 353). The major components of most capsicum species are capsaicin (69%), dihydrocapsaicin (22%), nordihydrocapsaicin (7%), homocapsaicin (1%), and homodihydrocapsaicin (1%), respectively (354). Capsaicin (8-methyl-*N*-vanillyl-*trans*-6-non-enamide) is a naturally occurring alkaloid derived from chilies. It is responsible for its hot pungent taste, characterized by its odorless fat-soluble compound that is rapidly absorbed via the skin.

*Lycium barbarum* polysaccharides and capsaicin have a protective effect in rats with dextran sulfate sodium-induced ulcerative colitis through anti-inflammation and antioxidation actions (355). Oral capsaicin has downregulated IL-6 and protein expression of TRPV1 and TRPA1 as well as increased SOD level and catalase activities (356). Besides, dietary capsaicin exhibited an anti-colitis effect in DSS-induced colitis wide-type (WT) mice by improving Na^+^ absorption, and reducing Cl^–^ level. It ameliorated intestinal inflammation by suppressing the hyperactivity of TRPV4 channels (357).

Capsaicin has an anticancer impact on HCT116 and LoVo cells (human colon cancer cells) through influencing cell cycle G0/G1 phase arrest and apoptosis, which was associated with an elevate of p21, BAX, and cleaved PARP. The capsaicin anticancer mechanism was attributed to the stabilization and activation of p53. It lengthened the half-life and boosted the transcriptional activity of p53 (358). Synergistic induction of both apoptosis and inhibition of cell proliferation was shown in HCT116, SW480, LoVo, Caco-2, and HT-29 (human CRC cells) that were treated with the combination of capsaicin and 3,3’-diindolylmethane. Also, these two compounds activated the transcriptional activity of NF-kB and p53 synergistically. The combination treatment stabilized nuclear p53 and up-or downregulated expression of several target genes that are downstream of NF-κB and p53 (359).

**FIGURE 5:**
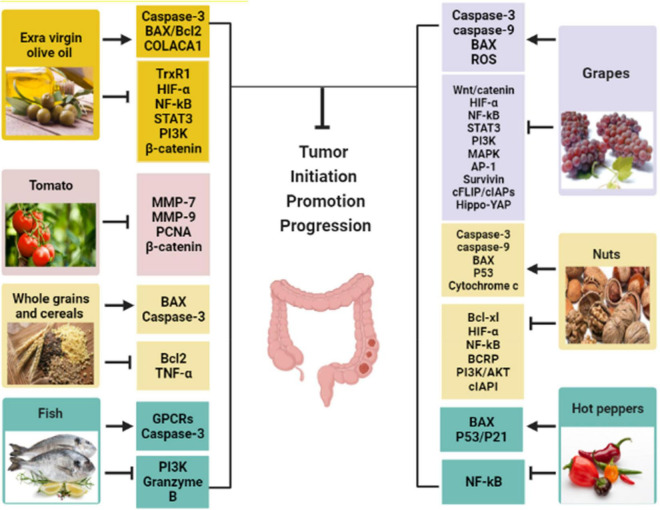
The Mediterranean diet components with their effects on cancer biomarkers. (→, activation; ⊤, inhibition; ROS, reactive oxygen species; TNF-α, tumor necrosis factor-alpha; IL, interleukin; MMP-9, matrix metalloproteinases-9; BAX, Bcl-2-associated X protein; Wnt, wingless-related integration; PI3K, phosphoinositide 3-kinase; PCNA, proliferating-cell nuclear antigen; TrxR1, thioredoxin reductase 1; COLACA1, colorectal cancer associated-1 gene; Bcl2, B-cell lymphoma 2; GPCRs, G protein-coupled receptors; MAPK, mitogen-activated protein kinase; AP-1, activator protein-1; c FLIP, cellular (FAAD-like IL-1β-converting enzyme)-inhibitory protein; cIAPs, cellular inhibitory of apoptosis proteins; BCRP, breast cancer resistance protein.

**FIGURE 6:**
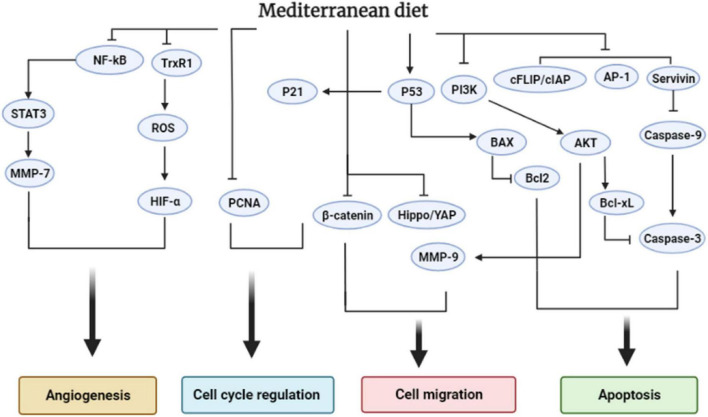
The effect of the Mediterranean diet on the cellular signaling pathways that regulate colorectal cancer progression.

Figure 5 demonstrated the seven MD components and their effects on cancer biomarkers and CRC development. Figure 6 displays the molecular mechanism of the MD in CRC.

Table 1 summarized the anti-inflammatory effects of all the discussed MD components. Table 2 summarized the anticancer effects of the MD components.

**TABLE 1:** Anti-inflammatory effects of the main Mediterranean diet components.

MD components	Active phytochemicals	Model of the experiment	Result of the study	References
Extra virgin olive oil	Hydroxytyrosol	DDS-induced colitis mice	↓ IL-6, IL-1β ↓ TNF-α ↓ Myeloperoxidase enzyme ↑ Probiotics	(49, 50)
		DDS-induced ulcerative colitis mice	↓ NLRP3 inflammasome ↓ IL-18, IL-1β ↓ Caspase-1	(50)
		Induced ulcerative colitis albino rats	↓Malondialdehyde, myeloperoxidase ↓ NO ↓ Mortality rate ↓ Disease activity index	(51)
	Oleuropein	Induced colitis rats	↓ IL-1β, IL-10 ↓ TNF-α ↓ COX-2 ↓ iNOS ↓TGF-β1 ↓ MCP-1 ↓ NF-κB	(67)
		DDS-induced colitis mice	↓ TNF-α ↓ IL-6 ↓ Neutrophil infiltration	(68)
Tomato	Lycopene	Ulcerative colitis rat	↓ IL-6, IL-1β ↓ TNF-α	(83)
		Ulcerative colitis rat	↓ NF-κB ↓TGF-β1 ↓ Caspase-3 ↓ GSH ↓ Catalase activity	(84)
		DSS-induced colitis mice	↓ TNF-α ↓ IL-1β ↓ iNOS ↓ TLR-4 ↓ *Bifidobacterium* and *Lactobacillus* ↓ Proteobacteria	(360)
		DSS-induced colitis mice	↓ Disease activity index score ↓ Colon length ↓ Catalase ↓ GSH-Px ↓ SOD	(85)
Onion	Quercetin	MC3T3-E1 preosteoblastic cell line	↓ NF-?B	(135)
		DSS-induced colitis mice	↓ GSH	(140)
		AOM/DSS-induced colon cancer mice	↓ LPO ↓ NO ↓ SOD ↓ G6PD ↓ GSH	(153)
Garlic	Allicin	Caco-2 cells	↓ p38 ↓ JNK	(185)
		AA-induced intestinal damage rats	↓ TLR4 ↓ NF-κB ↓ SCFAs	(185)
		TNBS-induced colitis Wistar rats	↓ TNF-α ↓ IL-6	(186)
Oregano	Carvacrol–thymol	Weaning-induced intestinal oxidative stress and inflammation piglets	↓ TNF ↑ *Lactobacillus*	(361)
	Carvacrol	Irinotecan-induced intestinal mucositis mice	↓ NF-κB ↓ COX-2 ↓ Oxidative stress ↓ NOx ↓ Malondialdehyde	(209)
Saffron	Crocin	colitis-associated CRC mice	↑ Catalase ↓ Malondialdehyde	(251)
		DSS-induced colitis mice	↓ COX-2 ↓ TNF-α ↓ NF-?B ↓ iNOS ↓ IL-6, IL-1β ↓ Nrf2	(240)
	Crocetin	TNBS-induced ulcerative colitis mice	↓ NO ↓ Neutrophil infiltration ↓ Lipid peroxidation ↓ NF-?B	(241)
Rosemary	Rosmarinic acid	AOM/DSS-induced colon cancer mice	↓ NF-?B ↓ STAT3	(263)
		DSS-induced colitis mice	↓ IL-6, IL-1β ↓ TNF-α ↓ iNOS ↓ COX-2	(264)
Whole grains and cereals	Arabinoxylan	DSS-induced colitis mice	↓ NF-?B ↓ NF-κBp65 ↓ TNF-α	(289)
	β-D-glucan	TNBS-induced colitis Sprague-Dawley rats	↓ IL-6, IL-10, IL-12 ↓ CRP ↓ COX ↓ PGE2 ↓ TXA2	(291, 292)
Fish	n-3 PUFA	DNBS-induced chronic colitis BALB/c mice	↓ TNF-α ↓ IFN-γ ↓ IL-17A	(306)
		Induced ulcerative colitis rats	↓ IL-1 ↓ Caspase-3 ↑ Bcl-2	(307)
Grapes	Resveratrol	HEK-293T cells and HeLa cells	↓ NF-κB ↓ TNF-α	(322)
		DSS-induced colitis mice	↓ IL-2, IL-1β, IL-6 ↓ IFN-γ ↓ TNF-α ↓ KC/GRO ↓ GM-CSF	(323)
Hazelnuts	β-sitosterol/stigmasterol	DSS-induced colitis C57BL/6J male mice	↓ Colon shortening ↓ Hemoglobin in feces ↓ NF-κB	(339)
	β-sitosterol	TNBS-induced colitis mice	↓ Colon shortening ↓ Myeloperoxidase ↓ TNF-α ↓ IL-1, IL-6 ↓ COX-2 ↓ NF-κB	(340)
		DDS-induced colitis male C57BL/6 mice	↓ IL-1, IL-6 ↓ TNF-α	(341)
Hot pepper	Capsaicin	DSS-induced colitis rats	↓ IL-6 ↓ TNF-α	(355)
		DSS-induced colitis rats	↓ IL-6 ↓ TRPV1, TRPA1 ↑ SOD ↑ Catalase	(356)
		DSS-induced colitis wide-type mice	↓ TRPV4	(357)

**TABLE 2:** Anticancer effects of the main Mediterranean diet components.

MD components	Active phytochemicals	*In vitro/In vivo*	Result of the study	References
Extra virgin olive oil	Hydroxytyrosol	*In vitro*	- Upregulation of the caspase-3 - Increase BAX/Bcl2	(54)
		*In vitro*	- Inhibition of TrxR1 - G1/S cell cycle arrest	(48)
		*In vitro/In vivo*	- Downregulation of epidermal growth factor receptors - Increased the expression of (COLACA1)	(77, 78)
	Oleuropein	*In vitro*	- Decreased HIF-α expression	(71)
		*In vivo*	- Repressed the activity NF-κB, STAT3, PI3K/Akt, and β-catenin	(72)
Tomato	Lycopene	*In vitro*	- Downregulated the MMP-7 expression - Hindered tumor development and tumor cell invasion	(86)
		*In vivo*	- Decrease the expression of PCNA and β-catenin proteins	(87)
Onion	Quercetin	*In vitro*	- G 2 cell cycle arrest	(148)
		*In vitro*	- Increased in the expression of CB1-R - Suppression of PI3K/Akt/mTOR signals	(145)
		*In vitro*	- Increased in Bax immunoreactivity	(153)
		*In vivo*	- Increased the apoptosis, cell proliferation and the expressions of p53 and Bax	(151)
		*In vivo*	- Reduced the LPO, NO, SOD, G6PD, GSH	(153)
Garlic	Allicin	*In vitro*	- Decreased in intracellular GSH levels	(187)
		*In vitro*	- Increased hypodiploid DNA content - Reduced levels of Bcl-2 - Increased levels of BAX, and cytochrome c	(188)
		*In vivo*	- Reduced the levels of phosphorylated STAT3 - Inhibited of Mcl-1, Bcl-2, and Bcl-xL	(180)
Oregano	Carvacrol	*In vitro*	- Increased in BAX - Decreased in Bcl-2, PARP, Survivin increased in the expression of caspase-3	(202)
		*In vitro*	- The G2/M cell cycle arrest - Reduced Bcl-2 - Phosphorylated p-ERK and p-Akt - Increased BAX and p-JNK expression	(213)
	Thymol	*In vitro*	Inhibition of JNK and p38	(214, 215)
Saffron	Crocin	*In vitro*	- Targeting MACC1 as a major causal metastasis-inducing gene	(248)
		*In vitro*	- Modulation of the Wnt/PI3K pathway - Regulating (CAT)and (MDA)	(251)
		*In vitro*	- Induction of apoptosis - Attenuation of the ratio of p-STAT3/STAT3	(250)
		*In vivo*	- Improved survival rate - Inhibited tumor development	(227).
	Crocetin	*In vitro*	- Cell cycle arrest - P21 induction - Increasing apoptosis - Lowering DNA repair capability	(249)
Rosemary	Rosmarinic acid	*In vivo*	- Reduced the rate of adenocarcinoma formation. - Upregulated TAS - Decreased the expression of IL-6 and MCP-1	(282)
		*In vivo*	- Suppressing tumor formation - Decreasing lipid peroxidation	(276)
Whole grains and cereals	β-glucan	*In vitro*	- Reducing Bcl2 expression - Upregulation of BAX and caspase-3 level	(298)
	β-glucan and quercetin	*In vivo*	- Reduced TNF-α - Increased the Parabacteroides - Downregulated Hmgcs2, Fabp2, Gpt	(297)
Fish	n-3 PUFA	*In vitro*	- Modulated COX metabolism - Reduced the PGE2 in tumors -increasing lipoxins and resolvins	(308)
		*In vitro*	- Downregulated:Wnt/ß-catenin pathway, the MAPK/ERK pathway, and the PI3K-PTEN system	(310)
		*In vitro*	- Activated pro-apoptotic signaling by interacting with GPCRs	(312)
		*In vitro*	- Inhibited signal transduction - Reducing cancer cell viability -encouraging apoptosis	(309)
Grapes	Resveratrol	*In vitro*	- Activation of P21 and P53 - Suppression of hippo–YAP - Inhibition of MAPK, (PI3K)/Akt, (NF-β) - Activating protein-1 (AP-1) HIF-1α - Signal transducer and activator of transcription 3 (STAT3)	(325–327)
		*In vitro*	- Upregulated BAX, cytochrome c, cleaved caspase-9, and caspase-3 - Reduced Bcl-2 expression levels	(328)
		*In vitro*	- Suppressing Wnt/-catenin signaling	(331)
		*In vitro*	- Downregulation of intracellular AKT1 and IL6 expression	(329)
Hazelnuts	β-sitosterol	*In vitro*	- Improved apoptosis via inducing p53 expression	(343)
		*In vitro*	- Decreased in Bcl-2 and mRNA -raised BAX protein and mRNA -suppressed cIAP1	(347)
		*In vitro*	- Restored oxaliplatin (OXA) - Disrupting the p53–MDM2 interaction - Silencing the NF-κB pathway	(344)
		*In vivo*	- Lowering PI3K/Akt expression, -promoting Bad activation - Decreasing Bcl-xl - Increasing cyto-c release	(362)
Hot pepper	Capsaicin	*In vitro*	- The G0/G1 cell cycle arrest - Elevated p21, BAX, and cleaved PARP.	(358)

## Role of the Mediterranean diet and its components in modulation of the gut microbiome

Several studies have shed the light on the role of the diet in the modulation and improvement of the gut microbiome (363). In particular, the MD which is recognized for its high plant-based food has shown a remarkable impact on gut microbiota profile (364, 365). It was found that adherence to MD has increased the presence of SCFA and fiber-degrading bacteria as well as reduced the presence of *E. coli* bacteria (365, 366). Besides, the positive impact of the MD on the gut microbiota content has been recognized by Garcia-Mantrana et al. (367), it was observed an improvement in Bifidobacteria abundance and higher production of SCFAs. Concurrent literature has described the beneficial modulation of the gut microbiome by the different components of the MD. Interestingly, the intervention treatment of hydroxytyrosol, a main phytochemical in EVOO, has modulated the gut microbiota leading to a lower density of inflammation-related microbes and enhancing the presence of probiotics (49, 50). Recently, Rocchetti et al. reported that oleuropein derived from olive leaf extract and EVOO has improved gut microbiota and potentiated the growth of bacteria associated with healthy metabolic markers (368). (360) reported the anti-inflammatory effect of lycopene in DSS-induced colitis mice. The study suggested that lycopene reduced the expression of TNF-α, IL-1β, TLR-4, and iNOS as well as modulated the gut microbiome by decreasing the density of proteobacteria and improving the presence of *Bifidobacterium* and *Lactobacillus* (360). Furthermore, allicin altered the structure of the gut microbiota and raised the number of beneficial bacteria. Koch and Lawson found that allicin suppresses the development of *Escherichia coli* and *Staphylococcus aureus* (369). Allicin (100 mg/kg/d) dramatically increased the relative abundance of *Ruminococcaceae*, *Clostridiales*, *Bacteroidales*, and *Facklamiaets* while decreasing the relative abundance of *Firmicutes*, *Corynebacteriales*, and *Lactobacillales* (370). Dietary supplementation with 100 mg/kg of carvacrol–thymol (CV–TH) (1:1) blend as animal feed for 14 days reduced weaning-induced intestinal oxidative stress and inflammation in piglets by decreasing tumor necrosis factor mRNA levels. It is worth noting that the CV–TH blend increased the population of *Lactobacillus* species and decreased the populations of *Enterococcus* and *E. coli* (361). Moreover, dietary fiber consumption enhances the creation and maintenance of a healthy, viable, and diversified colonic microbiota, acting as prebiotics. Prebiotics are ingredients that are resistant to gastric acidity and hydrolysis by enzymes (295). On the other hand, β-sitosterol maintained gut microbiota compositions leading to the production of beneficial metabolites including SCFAs that promote tumor apoptosis (362). As well, pungent food, particularly Capsaicin, has a positive action on gut flora, by decreasing the disease-causing enteric pathogens and encouraging the growth of useful bacteria (371).

## Chemopreventive effect of the Mediterranean diet and its components: Clinical studies

Despite the effectiveness of colonoscopy screening and recent improvements in cancer therapy, CRC remains one of the most prevalent and deadly types of cancer (372). Many studies have shown that a diet rich in fruits, vegetables, and tea is associated with lower rates of cancer, particularly colon cancer (373–375). In this context, a double-blind randomized clinical trial, case-control study, and meta-analysis revealed a substantial impact on colon disease and cancer patients. Fruit and deep-yellow vegetables, dark-green vegetables, onions, and garlic are moderately associated with a lower risk of colorectal adenoma, a precursor to CRC (376–378). Case-control studies generally revealed a lower risk of CRC with onion eating. In Argentina, the effect was more pronounced for consumption of a combination of garlic, onions, and pepper (379). In other case-control studies, the data points to a positive outcome. A food frequency questionnaire was used to analyze the intake of onions and garlic in a network of Italian and Swiss research that comprised 1037 cases and 2020 controls (380). The researchers discovered that onions and garlic were both protective against large bowel cancer. All levels of onion consumption were related to a lower risk of CRC. Also, Garlic usage at intermediate and high levels was linked to a lower risk of CRC. Several studies have indicated that flavonols, such as quercetin, can reduce the incidence of colon cancer with or without additional supplement therapy such as aspirin or NSAIDs (381–383). A dose-response meta-analysis revealed that an increase in dietary flavonols (such as quercetin) intake of 10 mg per day was significantly related to a reduced risk of CRC (383). Furthermore, taking 3.65 kg of garlic supplements per year for 2 years was connected with a lower incidence of colorectal adenoma, a precursor of CRC (375, 376). Epidemiological investigations of randomized controlled trials revealed that treatment of aged garlic extract reduced colon adenomas and CRC in patients with CRC via increasing NK cell activity (384). In a Japanese study, patients with both colorectal aberrant crypt foci (ACF) and colorectal polyps who were planning polypectomy had a double-blind, randomized controlled experiment to investigate the effectiveness of omega-3 FAs in humans. After a month of supplementation, EPA (2.7 g per day) was found to be more effective at inhibiting colorectal aberrant crypt foci than the placebo control group (385). Another clinical trial looked at how co-supplementing with vitamin D and omega-3 fatty acids affected inflammatory markers and the tumor marker CEA in chemotherapy-treated CRC patients. Eighty-one patients with stage I or stage II CRC were given two omega-3 fatty acid capsules and a 50,000 IU vitamin D soft gel once a week for 8 weeks. The findings demonstrated that, when compared to baseline, omega-3, vitamin D, and co-supplementation significantly reduced serum levels of TNF-, IL-1, IL-6, IL-8, and tumor marker CEA. In comparison to baseline, NF-kB activity was significantly reduced in the vitamin D and co-supplementation groups (386). Numerous clinical pilot investigations have demonstrated that resveratrol in high doses is generally safe. Twenty CRC patients received resveratrol before surgery, at doses of 0.5 g or 1.0 g taken orally for 8 days. According to the findings, resveratrol was well tolerated. In CRC resection tissue, resveratrol and its metabolites were discovered. Resveratrol (0.5 or 1.0 g) was sufficient to provide anticarcinogenic effects in colon cancers by reducing tumor cell proliferation by 5% (*P* < 0.005) (387) Furthermore, in nine patients with colon cancer and liver metastases, a daily injection of 5 g micronized resveratrol resulted in a 39% rise in cleaved caspase-3, a marker of apoptosis (388). In a double-blind, randomized, placebo-controlled study, tomato lycopene extract exhibited a chemopreventive effect in colon cancer patients (*n* = 56) via downregulation of insulin-like growth factor-1 levels (389). As well, an Italian case-control study confirmed that high adherence to MD can reduce CRC risk (390). However, more research involving human clinical studies is needed to prove the therapeutic effects of these phytochemical substances in the treatment of CRC.

## Future prospects

The future of the MD is rather unclear, and the MD’s health-protective qualities might be lost even before we completely realize the activity of the chemicals and the processes by which health results are attained. To maximize the potential health benefits, it is also essential to pay closer attention to the preservation of traditional foods and a faithful reflection on cultural traditions and the MD diet. Many studies have shown that high adherence to the Mediterranean pattern could significantly reduce the incidence of CRC. Hence, the recommendation of these diet patterns is usually as chemopreventive and in particular, cases can be applied as a complementary treatment to reduce tumor recurrence or protect from second tumors in recovered patients. However, more clinical research is required to determine the suitable and effective food patterns that can be administrated in CRC cases either for prevention or as a therapy. Besides, focusing on investigating the molecular mechanisms of MD components and their phytochemicals will be essential to upgrading the complementary therapies to the rank of established anticancer agents.

## Conclusion

The MD components are rich in phytochemicals with spectacular medicinal properties. It is believed that these components exert a nutritional synergy when consumed in combination. Many preclinical and clinical studies have demonstrated the cancer-preventive effects of the natural compounds involved in the MD patterns. Based on the collected facts in this review, these nutraceuticals could prevent CRC by either reducing inflammation or preserving a healthy microbiota in the intestine.

## Author contributions

All authors listed have made a substantial, direct, and intellectual contribution to the work, and approved it for publication.
